# Complete obliteration of a spontaneous pediatric vertebral arteriovenous fistula with patency of the parent vertebral artery: A case report

**DOI:** 10.1097/MD.0000000000017466

**Published:** 2019-10-11

**Authors:** Chang-wei Zhang, Ting Wang, Seidu A. Richard, Xiao-dong Xie

**Affiliations:** a Department of Neurosurgery, West China Hospital, Sichuan University, 37 Guo Xue Xiang Street, Chengdu, P. R. China; b Department of Medicine, Princefield University, Ghana, West Africa.

**Keywords:** angiography, balloon, endovascular, spontaneous, VAVFs

## Abstract

**Rationale:**

Vertebral arteriovenous fistulas (VAVFs) are depicted with anomalous connections between the vertebral artery, or its branches, and the adjacent venous system. Most VAVFs occur as a result of direct trauma during accidents, whereas others have iatrogenic origin.

**Patient Concerns:**

We report a case of 11-year-old male who presented with right limb weakness and walking instability.

**Diagnosis:**

Magnetic resonance angiography as well as digital subtraction angiogram (DSA) of the neck demonstrated a right VAVF. The cervical medulla was compressed by a dilated vein in vertebral canal. The blood supply of the fistula was from the right vertebral artery, whereas drainage was via epidural and paraspinal venous plexus.

**Interventions:**

We introduced the TransForm Occlusion Balloon Catheter into right vertebral artery, identified the VAVF, and occluded it with the balloon.

**Outcomes:**

We successfully obliterated the VAVF with patency of parent vertebral artery with a balloon. The symptoms of the patient were relieved after the procedure. Two years’ follow-up revealed no recurrence of the fistula. The patient is currently well.

**Lessons:**

Patency of the parent artery following obliteration a VAVF is still a challenge. Obliteration of the VAVF with a balloon while the parent vertebral artery is still patent is very possible.

## 1 Introduction

Vertebral arteriovenous fistulas (VAVFs) are depicted with anomalous connections between the vertebral artery, or its branches, and the adjacent venous system.^[1,2]^ Most VAVFs occur as a result of direct trauma during accidents, whereas others have iatrogenic origin.^[3]^ Interesting, VAVFs that do not occur as a result of trauma or iatrogenic origin are either congenital or spontaneous.^[3,4]^ The clinical presentations usually vary from asymptomatic to symptomatic with the most cardinal symptom being tinnitus. Also, vertigo and diplopia usually occur as a result of vertebrobasilar ischemia.^[3]^ The criterion standard radiological modality used in evaluating VAVFs is conventional catheter angiography.^[1,3,5]^ This modality is able to reveal all the anatomic characteristics of the VAVF as well as offer the accurate hemodynamics states, thereby aiding in planning interventional procedures. Nevertheless, computed tomographic angiography is also valuable in evaluating VAVFs.^[3,6]^ We present a pediatric case of VAVF in whom we successfully obliterated the VAVF with patency of parent vertebral artery.

## 2 Case presentation

**Figure 1:**
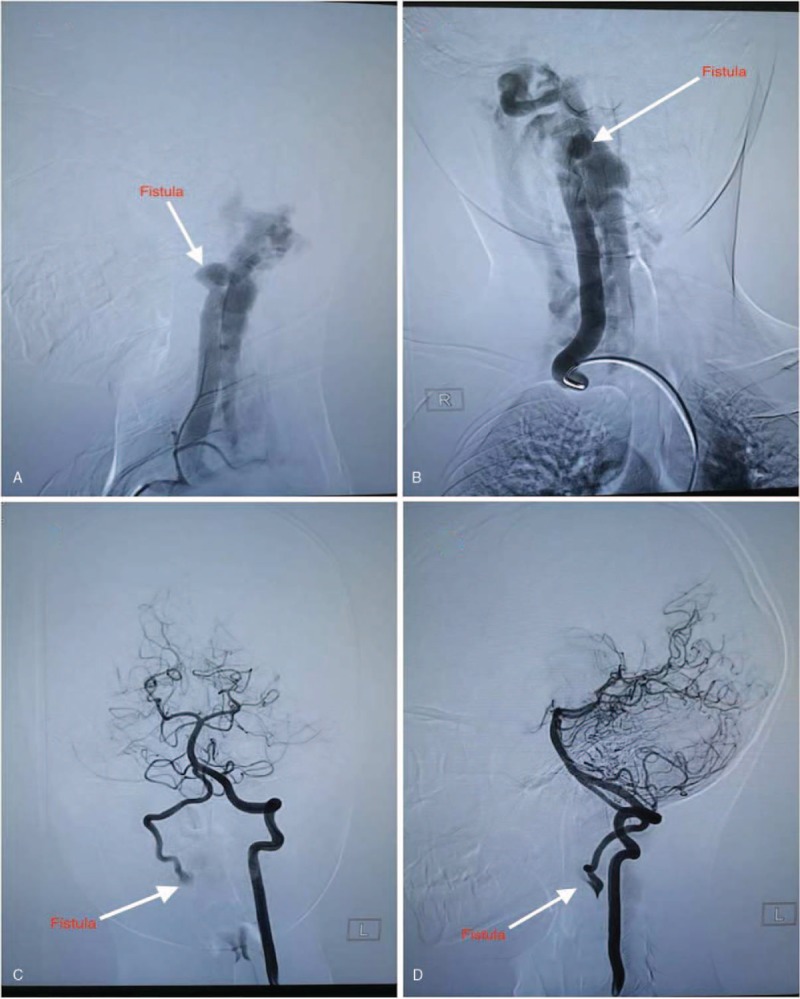
A and B are preoperative digital subtraction angiogram (DSA) images showing the lateral and anteroposterior views of right vertebral artery. C and D are preoperative DSA images showing the anteroposterior and lateral views of left vertebral artery.

**Figure 2:**
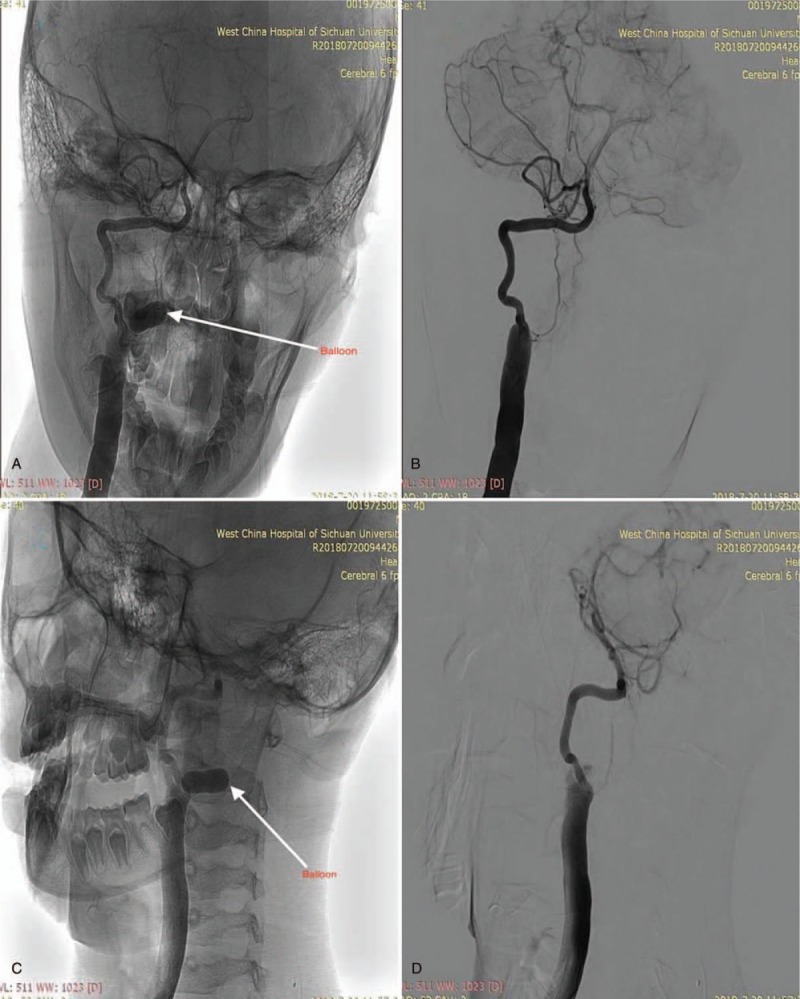
A and B are postoperative digital subtraction angiogram (DSA) images showing the anteroposterior view of right vertebral artery. C and D are postoperative DSA images showing lateral view of right vertebral artery.

**Figure 3:**
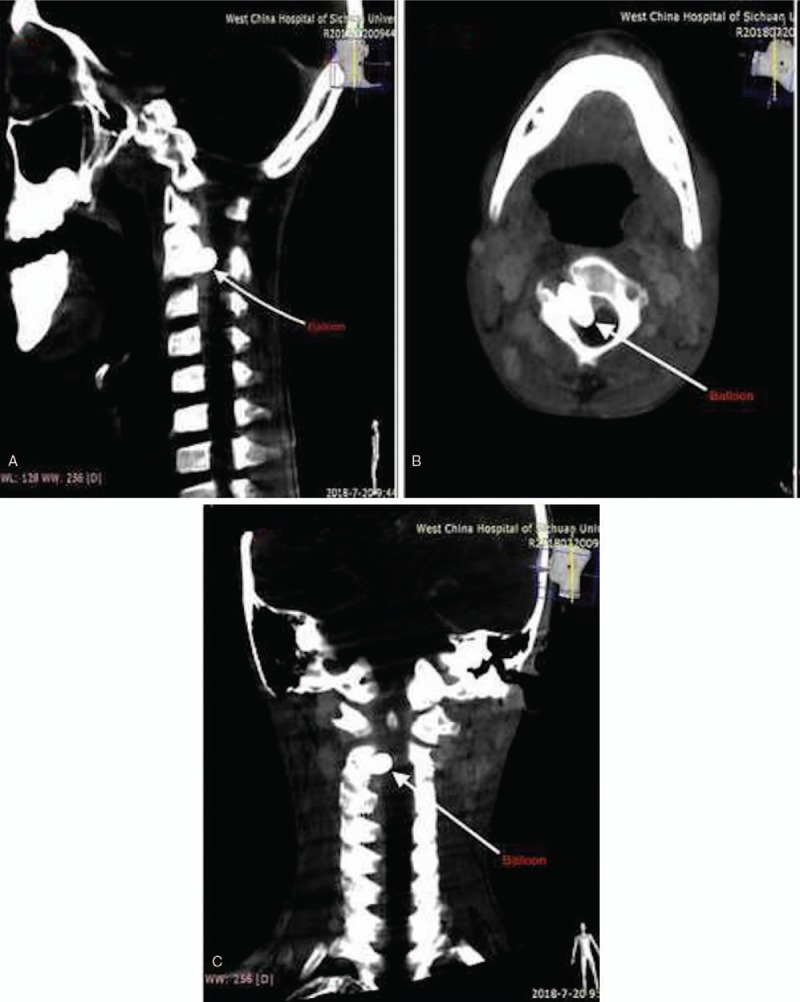
A, B, and C are postoperative magnetic resonance imaging images showing occlusion of the fistula with the balloon.

An 11-year-old male presented with right limb weakness and walking instability. We noticed a pulsating swelling on the right lateral side of the neck. The swelling was around the C1 and C2 vertebral levels. Auscultation on the swelling revealed a bruit. There was no history of trauma before this presentation. He has never had a central line passed before or cannulation of veins in the neck. His medical history was unremarkable. He was initially seen and managed in a local hospital before his referral to our facility. Magnetic resonance angiography done at our facility (West China Hospital) showed a fistula with a wide neck between the right distal vertebral artery and paraspinal venous plexus. This prompted us to do a digital subtraction angiogram (DSA). On DSA, we noticed a high-flow fistula with total antegrade blood flow from the V3 segment of the vertebral artery (Fig. 1A–D). No further arterial supply was found except the vertebral artery. The venous drainage was via a complex venous plexus of epidural and paraspinal veins. We noticed during our evaluation that preservation of both the distal and proximal vertebral artery via endovascular obliteration would not result into severe complications owing to none contrast filling at the right distal vertebral artery. We also observed that the contralateral compensatory blood supply to the basilar artery was adequate. However, posterior cerebellar and/or spinal infarct risk and the fact that the patient already had neurological defect, prompted us to preserve the vertebral artery. Therefore, endovascular approach with balloons was the most suitable treatment option. We introduced the TransForm Occlusion Balloon Catheter into right vertebral artery, identified the VAVF, and occluded it with the balloon. We successfully obliterated the VAVF with patency of parent vertebral artery (Fig. 2A–D). The symptoms of the patient were relieved immediately after the procedure. Postoperative magnetic resonance imaging revealed occlusion of the fistula with the balloon (Fig. 3A–C). Two years’ follow-up revealed no recurrence of the fistula. The patient is currently well.

## 3 Discussion

VAVFs are depicted with anomalous connections between the vertebral artery, or its branches, and the adjacent venous system.^[2,7]^ Most VAVFs occur as a result of direct trauma during accidents, whereas others have iatrogenic origin. Iatrogenic VAVFs can occur as a result of direct angiographic puncture of the vertebral artery or as a result of struggles in cannulating the internal jugular vein during insertion of a central line.^[2]^ Our patient was a child with spontaneous VAVF. Interestingly, our patients did not have fibromuscular dysplasia or neurofibromatosis, although majority of spontaneous VAVFs occurred in patients with these anomalies.^[7,8]^

To accurately and efficiently diagnose as well as manage VAVFs cases, Lasjaunias divided them into upper cervical and lower cervical.^[7,9]^ The upper cervical VAVFs usually encompass lesions at Cl-2 level, whereas lower cervical VAVFs encompass lesions below this level. Most often, the upper cervical VAVFs usually derived their blood supply from the occipital artery through an anastomoses with the vertebral artery.^[7,9]^ Also, for a better safeguarding of the vertebral artery, Lasjaunias^[9]^ further divided the VAVFs in the upper cervical group into segmental and intersegmental. Nevertheless, in most cases, the venous drainage is usually channeled to the suboccipital and vertebral plexus.^[7]^

The single most important sign used in diagnosing VAVFs is a bruit.^[7]^ Also, symptoms like tinnitus or even heart failure from shunting may be obvious. Nevertheless, in cases with high-flow fistulae, symptoms such as hemorrhage, steal phenomena, intracranial venous hypertension, or embolic stroke may also occur.^[10]^ Furthermore, cervical radiculopathy may also occur when there is a direct compression of the nerve roots by the draining veins.^[7,11]^ Myelopathy has been reported in some cases. This often occurs as a result of mass effect from dilated veins in the epidural space or an epidural bleed. Myelopathy may also be secondary to venous hypertension.^[7,12]^ Conventional or selective catheter angiography is the “criterion” standard modality used to diagnose VAVFs.^[1,4,7]^

Endovascular techniques are the preferred management modality of VAVFs. These techniques usually utilize detachable balloons, stent grafts, as well as detachable coils as embolic materials.^[1,2]^ Most often, the principal aim of management is occlusion of the fistula as well as conservation of the vertebral artery. This is often very difficult to achieve. Nevertheless, during the procedure, the surgeon should as much as possible prevent proximal vessel ligation to avoid vertebrobasilar inadequacy, as this usually increases retrograde flow.^[7,9]^ Although various agents as well as treatment modality have been proposed for the management of VAVFs, balloons are preferred since precise positioning of the embolic agent can be done.^[5,7]^ Besides, other embolic agents may be used to supplement the closure in cases of partial closures with balloons. In our case, we achieved total occlusion of fistula with patency of the parent artery. Usually, the use of balloons may not be feasible, particularly from the contralateral artery, if there is obvious tortuosity or narrowing of the arterial lumen. The most suitable treatment modality after careful evaluation of our patient was the use of a balloon.

Some authors have used the contralateral approach to achieve satisfactory results. In this approach, they used coils to occlude the vertebral artery just distal to the fistula. The traditional surgical procedure usually involves direct surgical ligature of punctiform fistula.^[1]^ Nevertheless, during surgical intervention, the surgeon obtains a bypass venous graft from the subclavian to reconstruct the distal vertebral artery for large, complex fistulas with several arterial feeders.^[1,5]^ The major drawbacks with surgical intervention are usually steal flow symptoms, massive hemorrhages, and injuries to surrounding structures.^[1,5]^ The use of endovascular stent-graft repair is not much as compare to other agents.^[13–15]^ These devices are best substitutes for the management of VAVF because they are able to preserve vessel patency. Nevertheless, the current stent-graft available is very rigid and not appropriate for vessels that are tortuous.^[1,13,14]^

Endovascular treatment with a detachable balloon to occlude the fistula is the best treatment modality as compared to microsurgical ligation, coil embolization, or stent-grafting.^[1]^ Several authors have demonstrated that detachable balloons are most appropriate for occlusion of fistulas. They argue that these balloons can be repeatedly inflated and deflated before their detachment so that exact position and ideal occlusion of the fistula can be attained.^[1,12,16]^ Regardless of which ever treatment modality that is adapted by the surgeon, evaluation of the fistula and parent vessel anatomy, hemodynamics and flow (high or low) is key to attaining successful fistula occlusions.^[1,17]^

## 4 Conclusion

We present a case of a VAVF in a pediatric patient in whom we used a balloon to completely obliterate the shunt with patency of the parent vertebral artery. Successful obliteration of the fistula with patency of parent vertebral artery has not been achieved in all reported cases in literature. The symptoms of the patient were relieved after the procedure and 2 years’ follow-up revealed now recurrence of the fistula.

## Author contributions

**Conceptualization:** Chang Wei Zhang, Ting Wang, Seidu A. Richard, Xiao Dong Xie.

**Data curation:** Chang Wei Zhang, Ting Wang, Seidu A. Richard.

**Formal analysis:** Ting Wang, Seidu A. Richard, Xiao Dong Xie.

**Funding acquisition:** Chang Wei Zhang, Xiao Dong Xie.

**Investigation:** Ting Wang.

**Methodology:** Seidu A. Richard.

**Supervision:** Chang Wei Zhang, Xiao Dong Xie.

**Writing – original draft:** Seidu A. Richard.

**Writing – review & editing:** Chang Wei Zhang, Ting Wang, Seidu A. Richard, Xiao Dong Xie.
